# A new upper limit to the field‐aligned potential near Titan

**DOI:** 10.1002/2015GL064474

**Published:** 2015-06-18

**Authors:** Andrew J. Coates, Anne Wellbrock, J. Hunter Waite, Geraint H. Jones

**Affiliations:** ^1^ Mullard Space Science LaboratoryUniversity College LondonLondonUK; ^2^ Centre for Planetary Sciences at UCL/BirkbeckLondonUK; ^3^ Southwest Research InstituteSan AntonioTexasUSA

**Keywords:** ionosphere, Titan, ambipolar field, escape

## Abstract

Neutral particles dominate regions of the Saturn magnetosphere and locations near several of Saturn's moons. Sunlight ionizes neutrals, producing photoelectrons with characteristic energy spectra. The Cassini plasma spectrometer electron spectrometer has detected photoelectrons throughout these regions, where photoelectrons may be used as tracers of magnetic field morphology. They also enhance plasma escape by setting up an ambipolar electric field, since the relatively energetic electrons move easily along the magnetic field. A similar mechanism is seen in the Earth's polar wind and at Mars and Venus. Here we present a new analysis of Titan photoelectron data, comparing spectra measured in the sunlit ionosphere at ~1.4 Titan radii (*R*_T_) and at up to 6.8 *R*_T_ away. This results in an upper limit on the potential of 2.95 V along magnetic field lines associated with Titan at up to 6.8 *R*_T_, which is comparable to some similar estimates for photoelectrons seen in Earth's magnetosphere.

## Key Points

- Photoelectrons are seen throughout the Saturn system including at Titan
- Energy peaks in Titan's ionosphere and tail determine field‐aligned potential
- Field‐aligned potential is 2.95 V; this limits the escape flux from this process

## 1 Introduction

The Saturn system is one of the most complex in the solar system, and since 2004 the Cassini orbiter has been exploring the planet, rings, icy satellites, magnetosphere, and Titan. One of the interesting aspects of the system is the neutral and plasma environment. The neutral particle density has been found to be much higher than the plasma density in Saturn's inner magnetosphere [*Esposito et al*., 2005; *Young et al*., 2005]. The neutral particle source is principally Enceladus orbiting at 4 Saturn radii (*R_S_*) with its geysers of gas and ice grains emerging from fractures near the south pole [*Dougherty et al*., 2006; *Porco et al*., 2006; *Waite et al*., 2006]. Particles originating at Enceladus even reach the orbit of Titan [*Smith et al*., 2009; *Thomsen et al*., 2010]. Neutral particles are also important near Saturn's rings due to the interaction of high‐energy particles with the ring particles [*Johnson et al*., 2010], which form an additional neutral source. Although weak exospheres have been discovered at Rhea [*Teolis et al*., 2010] and Dione [*Tokar et al*., 2012], the associated neutral densities are very low compared with those near Titan or Enceladus. However, Titan orbiting at 20 *R_S_* remains a fascinating object with a significant, nitrogen‐ and methane‐rich atmosphere including complex organics [e.g., *Strobel et al*., 2010, and references therein] and associated plasma environment that have been studied during many encounters by the Cassini spacecraft.

In all of these locations, the neutral particles may be ionized, producing new ions and photoelectrons. The photoelectrons have particular energies determined by the solar spectrum and the ionization potential of the target gas. The difference between the solar photon energy and the ionization potential gives the energy. In this process, the prominent 30.4 nm line in the solar spectrum produces peaks in the 20–30 eV region [see *Nagy and Banks*, 1970; *Mantas and Hanson*, 1979; *Fox and Dalgarno*, 1979; *Coates et al*., 2011, and references therein]. The newly born ions and the photoelectrons then interact with their plasma environments. At Titan, photoelectrons are observed directly in the sunlit ionosphere and at several Titan radii along the Titan tail [*Coates et al*., 2007a].

Previous modeling work had already recognized the likely importance of photoelectron transport out of Titan's ionosphere [*Gan et al*., 1992, 1993]. Our previous observations (briefly reviewed below) and the new observations presented here confirm this importance and explore new aspects.

In this paper, we first briefly summarize the observations of photoelectrons in the Saturn system. We then present new spectra taken in Titan's ionosphere and at several Titan radii along the tail. We use the observed energy of the peak associated with the 30.4 nm solar radiation, to determine an upper limit for the magnetic field‐aligned potential and conclude that a polar wind‐style escape is likely taking place.

## 2 Instrumentation

The Cassini Plasma Spectrometer (CAPS) Electron Spectrometer (ELS) [*Young et al*., 2004; *Linder et al*., 1998] is a top‐hat electrostatic analyzer which produced a 63‐point energy spectrum every 2 s, with Δ*E*/*E* ~ 17% measured [*Linder et al*., 1998] and ~15.5% steps in this energy range (from applied voltage; matched at higher energies). It has a 160°×5° field of view divided into eight angular sectors. The three adjacent energy bins in the region of interest for ionospheric photoelectrons at Titan cover the ranges, at full width at half maximum in the response curve, 17.62–20.90 eV, 20.32–24.10 eV, and 24.10–28.58 eV; these bins are from hereon referred to via their center energies, 19.26, 22.21, and 26.34 eV which are accurately known from ground calibration. Spacecraft potential affects the measurements from ELS, and in the Titan ionosphere this potential is generally slightly negative (~0 to −2 V) [e.g., *Wahlund et al*., 2005; *Coates et al*., 2007a; *Crary et al*., 2009; B. Magee, personal communication, 2012; K. Ågren, personal communication, 2012].

## 3 Photoelectrons in the Saturn System

### 3.1 Main Rings

*Coates et al*. [2005] presented observations of thermal (~0.6–100 eV) electrons observed near Saturn's main rings during Cassini's Saturn Orbit Insertion on 1 July 2004. They observed that the energy spectrum over the Cassini division and the A ring was most likely related to photoelectron production in a ring atmosphere/ionosphere. Together with ion measurements over the rings [*Tokar et al*., 2005], the presence of a photoelectron peak in the data was taken as evidence for a ring atmosphere and ionosphere. This had been predicted by models [e.g., *Ip*, 1986] and further modeling was performed [e.g., *Bouhram et al*., 2006].

### 3.2 Inner Magnetosphere

*Schippers et al*. [2009] presented observations of characteristic photoelectron peaks in the inner regions of the Saturn magnetosphere during low‐latitude orbits of the Cassini spacecraft. They found peaks at about 20 eV and 42 eV after removal of signals associated with penetrating radiation. They used pitch angle information to assess the near‐equatorial source location of these photoelectrons and a simple model of chemistry in order to further support the interpretation as photoelectrons. They estimated the density of these photoelectrons to be ~1% of the total electron density. The main features of the observations were confirmed by modeling [*Cravens et al*., 2011].

### 3.3 Enceladus Plume

*Coates et al*. [2013] summarized the plasma environment in the plume of Enceladus as containing cold magnetospheric electrons, negative and positive water clusters [*Coates et al*., 2010a; *Tokar et al*., 2009], charged nanograins [*Jones et al*., 2009; *Hill et al*., 2012], and “magnetospheric photoelectrons” produced from ionization of neutrals throughout the magnetosphere near Enceladus ([*Schippers et al*., 2009] as mentioned above). *Coates et al*. [2013] additionally discovered photoelectrons produced in the plume ionosphere itself. Such plume photoelectrons are seen at all Enceladus encounters where electron spectra are available. The plume photoelectron population is warmer than the ambient plasma population, providing an additional ionization source in this region.

### 3.4 Titan

*Coates* [2009] presented data from the TA encounter, which was the first close flyby of Titan. One aspect of the data was the photoelectron peak at 24.09 eV (after correcting for spacecraft potential) seen in the sunlit ionosphere which is associated with primary photoelectrons from the ionization of nitrogen due to the prominent solar radiation line at 30.4 nm [*Coates et al*., 2011]. This was subsequently seen in all encounters that traversed the sunlit Titan ionosphere. They also discussed the results of *Coates et al*. [2007a], who presented results from ELS during the downstream flyby of Titan on 26 December 2005 (T9). The electron data showed a split signature in the distant tail with two principal intervals of interest outside the nominal corotation wake. Interval 1 showed direct evidence for ionospheric plasma escape at several *R*_T_ in Titan's tail. Interval 2 showed a complex plasma structure, a mix between plasma of ionospheric and magnetospheric origin. In the case of interval 1, clear photoelectron peaks were seen. Since neutral nitrogen densities are very low in Titan's tail, these photoelectrons would have been created in Titan's dayside ionosphere where neutral densities are high enough. Therefore, the presence of photoelectron peaks indicates a magnetic connection between the production point (in the sunlit ionosphere) and the spacecraft location. They suggested a mechanism for plasma escape based on ambipolar electric fields set up by suprathermal ionospheric photoelectrons. This mechanism was similar to that of the “polar wind” at Earth [e.g., *Ganguli*, 1996] and has also been suggested to enhance plasma escape at Mars [*Coates et al*., 2011] and at Venus [*Coates et al*., 2011; *Tsang et al*., 2015; *Coates et al*., 2015].

In the polar wind mechanism, the relatively energetic photoelectrons move easily along the magnetic field, the charge separation sets up a parallel ambipolar electric field *E*_∥_, which acts to extract ions from the ionosphere. The approximate strength of the ambipolar electric field can be written as E∥∼1Ne∇Pe, where *N_e_* and *P_e_* are the density and pressure of the electrons [see *Yau et al*., 2007, and references therein]. The magnitude of the potential is thus controlled by the average electron kinetic energy.

*Wellbrock et al*. [2012] described additional photoelectron peak observations at large distances from Titan, on three encounters (T15, T17, and T40) and discussed the observed tail structures. They inferred that the distant photoelectrons traveled to the observation sites by means of a magnetic connection from their production point at lower altitudes in the dayside ionosphere. This idea is supported by results of hybrid modeling [*Wellbrock et al*., 2012]. Thus, photoelectrons may be used as tracers of magnetic field lines. The T17 example was a particularly interesting one as photoelectrons were seen almost continuously from ~12,000 km altitude on Cassini's inbound trajectory to soon after closest approach [*Wellbrock et al*., 2012].

*Coates et al*. [2012] examined the data from three more distant tail flybys, T9 as discussed above, T75 (at a similar downtail distance to T9), and T63 (at about half this distance). All three traversed the nominal corotation wake but at different local times, and all three contained a similar split tail signature, with ionospheric plasma, as indicated from the electron energy spectrum seen flowing along the magnetotail, again transported along draped magnetic field from the sunlit ionosphere. They estimated escape rates along the tail based on observations of ionospheric electrons and ions.

## 4 Determination of the Field‐Aligned Potential at Titan

We now examine the photoelectron peak energy seen at several traversals of Cassini through the sunlit ionosphere and compare with the photoelectron peak energies seen in the distant tail crossings, T9, T75, and T63.

We examine data from a total of 18 flybys through the sunlit ionosphere, shown in Table 1. These dates were chosen to be flybys (selected between TA and T71) through the ionosphere at a range of solar zenith angles, latitudes, and Saturn local times, to be a representative sample of flybys. We have also included the flybys where more distant photoelectrons were measured as studied by *Wellbrock et al*. [2012]; we also include a spectrum (indicated as T17A) from the distant tail region they identified on the T17 encounter. The distant tail flybys studied by *Coates et al*. [2012] are also shown in the table.

**Table 1:** Summary of the Cassini Titan Flybys Studied Here^a^

		CA		Altitude	SLT	Lat	SZA	Spectrum Time	Spectrum Altitude Range	PE Peak Energy
Flyby	Date	(hh:mm)	DOY	(km)	(hh:mm)	(°N)	(deg)	(hh:mm:ss)	(km)	(eV)
TA	26 Oct 2004	15:30	300	1,176	10:36	39.4	92.9	15:26	1,439–1,330	22.21
T15	2 Jul 2006	09:21	183	1,906	21:13	Tail	Tail	09:12	2,806–2,631	22.21
T17	7 Sep 2006	20:17	250	1,000	02:19	21.7	41.8	20:12:30	1,302–1,185	22.21
T18	23 Sep 2006	18:58	266	960	02:17	70.5	89.6	18:53	1,486–1,333	19.26–22.21
T19	9 Oct 2006	17:30	282	980	02:14	61.4	81.3	17:34	1,224–1,354	22.21
T20	25 Oct 2006	15:57	298	1,042	02:11	8.6	29	16:02	1,271–1,400	22.21
T23	13 Jan 2007	08:38	13	1,004	01:57	33	54.3	08:33	1,474–1,329	22.21
T36	2 Oct 2007	04:41	275	973	11:29	−59.2	71.8	04:46:30	1,233–1,374	22.21
T39	20 Dec 2007	22:58	354	970	11:21	−69.1	60.4	23:02	1,274–1,425	22.21
T40	5 Jan 2008	21:30	5	1,015	11:19	−11.1	36.6	21:25	1,509–1,353	22.21
T41	22 Feb 2008	17:32	53	1,003	11:13	−33.2	27.6	17:26	1,646–1,469	22.21
T42	25 Mar 2008	14:27	85	1,001	11:07	−28.9	24.3	14:22:30	1,496–1,502	22.21
T43	12 May 2008	10:01	133	1,002	10:59	17.8	36	09:55	1,822–1,625	22.21
T48	5 Dec 2008	14:25	340	961	10:22	−11	24.5	14:20	1,533–1,366	22.21
T49	21 Dec 2008	12:59	356	972	10:19	−47.5	81.3	12:55	1,389–1,244	22.21
T61	25 Aug 2009	12:50	237	961	21:42	−18	92.4	12:54:30	1,098–1,202	22.21
T64	28 Dec 2009	00:16	362	952	16:57	82.7	86.5	00:20	1,101–1,208	22.21
T65	12 Jan 2010	23:10	12	1,074	16:56	−82.2	95.2	23:12:30	1,132–1,205	22.21
T71	7 Jul 2010	00:22	188	1,004	16:03	−56.3	83	00:18	1,357–1,232	22.21
T9	26 Dec 2005	18:59	360	10,411	03:03	Tail	Tail	18:36	12,575–12,412	22.21
T75	19 Apr 2011	05:00	109	10,053	14:14	Tail	Tail	04:21:40	15,425–15,200	22.21
T63	12 Dec 2009	01:03	346	4,850	16:57	Tail	Tail	00:54	5,463–5,344	22.21
T17A	7 Sep 2006	20:17	250	1,000	02:19	Tail	Tail	19:36	11,816–11,500	22.21

a Closest approach (CA), Day of Year (DOY), Saturn Local Time (SLT), Latitude (Lat), and Solar Zenith Angle (SZA) are shown with other parameters for each flyby (except for intermediate and distant tail crossings, marked “Tail” in the Lat and SZA columns). The start time for each 1 min average electron spectrum (plotted in Figure 3) is shown, with the peak energy of the ~22 eV peak determined from each spectrum.

**Figure 1:**
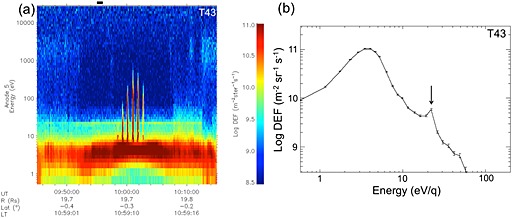
(a) ELS anode 5 data taken during the T43 flyby. The ionospheric photoelectron peak at 22.1 eV is prominent during the flyby. The dashed white line labeled “CA” indicates the closest approach time at an altitude of 1002 km. (b) Electron spectrum (1 min average) beginning at 09:55 UT (interval shown by black horizontal bar at the top of Figure 1a). The error bars are based on Poissonian statistics. The arrow indicates the center of the 22.21 eV bin.

Figure 1a shows a CAPS ELS spectrogram from the T43 flyby, as an example of a pass through the sunlit ionosphere. Closest approach (CA) is toward the center of the plot. The broad peak below 10 eV is the main suprathermal ionospheric population. The vertical spikes and peaks near 10:00 UT are due to negative ions [*Coates et al*., 2007b, 2009, 2010b; *Wellbrock et al*., 2013]. The weak flux at ~2 keV is the remaining electron population from Saturn's magnetosphere. The prominent line across most of the plot at ~22 eV is due to primary photoelectrons associated with the ionization of nitrogen by the 30.4 nm peak in solar EUV radiation.

In Figure 1b we show an averaged (over 1 min) electron energy spectrum from T43, at the time shown by the horizontal black bar in Figure 1a. The broad peak at ~4 eV and the peak at ~22 eV are clearly visible with the latter highlighted with an arrow. Examining the energy of this peak in detail, the nearest central energy bins sampled by ELS (with an energy resolution and bin size of Δ*E*/*E* ~ 17%) are 19.26, 22.21, and 26.34 eV. The actual peak is clearly at 22.21 eV in this spectrum. The anticipated energy for this peak is actually 24.09 eV [see *Coates et al*., 2011]. The measured energy of the peak (22.21 eV) is additionally determined by the energy bins sampled and by the negative spacecraft potential and is consistent with the 24.09 eV expectation. Figure 1b also shows a reduction in electron flux at ~50–60 eV, again a characteristic signature in photoelectron fluxes due to a reduction in the solar spectrum near 16 nm [e.g., *Nagy and Banks*, 1970; *Mantas and Hanson*, 1979; *Fox and Dalgarno*, 1979].

**Figure 2:**
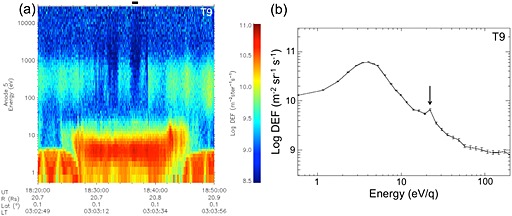
(a) ELS data taken during interval 1 [*Coates et al*., 2007b, 2012] of the T9 flyby through the Titan tail. The ionospheric photoelectron 22.1 eV peak is seen intermittently; Cassini is 6.8–5.4 *R*_T_ downstream from Titan at this time. (b) Electron spectrum (1 min average) beginning at 18:36 UT (interval shown by black horizontal bar in Figure 2a). The error bars are based on Poissonian statistics. The arrow indicates the center of the 22.21 eV bin.

In Figure 2a we show data from T9, one of the distant tail flybys, concentrating on interval 1 (~18:24–18:44 UT, corresponding to ~6.8–5.4 *R*_T_ along the tail). Here ionospheric plasma is seen, transported from the sunlit ionosphere [*Coates et al*., 2007a]. Ionospheric plasma is again seen as the broad peak at ~10 eV and below, with the magnetospheric electrons at ~100–1000 eV in this case. As this encounter is well along Titan's tail, and the sign of any field‐aligned potential would accelerate positive ions from the ionosphere, no negative ions are seen here, although a ~22 eV electron peak is again seen in the data several times in this interval. A related simultaneous ion population was reported by *Coates et al*. [2007a], and this was taken as evidence for escape driven by ambipolar electric fields.

Figure 2b again shows a 1 min averaged electron spectrum taken at the time of the horizontal black bar in Figure 2a. We again observe a ~4 eV ionospheric electron peak, as well as the second peak in the 22.21 eV energy bin, as in Figure 1b. Similarly, the spectrum shows a reduction (though less pronounced than in Figure 1b) at ~50–60 eV again, consistent with the ionospheric electron interpretation.

**Figure 3:**
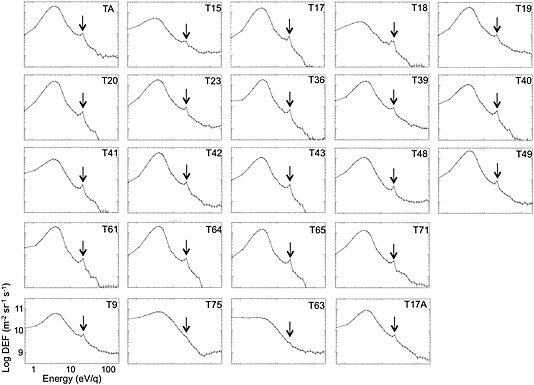
Electron spectra from all the Titan ionosphere flybys studied in this paper (see Table 1). The axes for each plot are shown in the T9 example. The bottom four plots are from Cassini's distant tail encounters T9, T75, T63 [*Coates et al*., 2012], and T17A [*Wellbrock et al*., 2012]. The error bars are based on Poissonian statistics. The arrow indicates the center of the 22.21 eV bin.

Figure 3 shows spectra from all of the intervals identified in Table 1, with the bottom four plots from the distant tail flybys. The spectra and the peak locations identified from them (shown in Table 1) are almost all in the 22.21 eV energy bin. In the case of T75 and T63, the 22.21 eV feature is an inflection rather than a peak, at the same observed energy. The 22.21 eV energy bin is indicated in all the panels.

## 5 Discussion

The results presented in the previous section show that the energy of the photoelectron peak is within the same energy bin of ELS for almost all of the passes through the sunlit ionosphere we have studied and for all of the distant tail passes. As the spacecraft is immersed in ionospheric plasma for all of the intervals studied, we may assume that the spacecraft potential is relatively low, typically ~0 to −2 V as mentioned above. Furthermore, the spacecraft potential does not change significantly during different encounters as plasma conditions, particularly the ionospheric electron temperature that would drive the spacecraft potential [e.g., *Whipple*, 1981], are very similar for all the passes studied. Inspection of spacecraft potential data from the RPWS Langmuir probe for the intervals studied here indicate that the potential is indeed between 0 and −2 V for the intervals studied [*Modolo et al*., 2007; K. Ågren, personal communication, 2012]. A negative spacecraft potential means that the measured electron energy is lower than the electron actual energy because the incoming electrons are retarded in traveling through this potential drop, and an energy equivalent to the potential needs to be added to the observed electron energy, increasing its magnitude. The resulting spacecraft potential corrected energy range of the measured photoelectron peak in the 22.21 eV energy bin therefore still includes the expected value of 24.09 eV due to the ionization of nitrogen by the intense solar radiation at 30.4 nm.

The T17 and T17A spectra are particularly interesting as the peak is clearly in the 22.21 eV bin for both. As mentioned earlier, photoelectrons were seen almost continuously along the tail during this encounter, with no change in the peak energy bin except a brief interval with the peak just in the 19.26 eV bin.

As the energy of the photoelectron peak is within the same energy bin of ELS for almost all of the passes, we conclude that any field‐aligned potential (up to the maximum spacecraft distance from Titan reported here, i.e., up to 6.8 *R*_T_) is less than the difference between the bin energies between the 19.26 and 22.21 eV energy bins of the ELS, i.e., 2.95 V. In fact the field‐aligned potential certainly must be less than this, as the negative spacecraft potential already modifies the peak energy of 24.09 eV further into the 22.21 eV bin range. The 2.9 V is thus clearly an upper limit for the field‐aligned potential up to the measurement position.

As mentioned above, the relatively energetic photoelectrons are more mobile along magnetic field lines than the ions and may set up an ambipolar electric field analogous to the polar wind at Earth [*Ganguli*, 1996, and references therein] and also relevant to processes at Mars [e.g., *Coates et al*., 2011] and Venus [e.g., *Hartle and Grebowsky*, 1995]. There have been several estimates of the field‐aligned potential related to the terrestrial polar wind.

Estimates of ~1–1.7 eV for the potential difference along a terrestrial field line were modeled by *Lemaire and Scherer* [1971], see also *Yau et al*., [2007]. Other authors have suggested that the energy of the suprathermal electrons may affect the field‐aligned potential and that tens of eV may even be possible [*Axford*, 1968], which were also observed at times (~13 eV by *Winningham and Gurgiolo* [1982], consistent with ~10 eV modeled by *Tam et al*. [1995]). However, electron measurements at Earth using the GEOS‐1 satellite were interpreted as a similar ionospheric photoelectron peak and a shoulder at ~60 eV [*Coates et al*., 1985]. These measurements imply that the maximum field‐aligned potential between the sunlit ionosphere and the observation point was <2 V at this time in the terrestrial case. A similar observation was recently reported with the Cluster spacecraft [*Fazakerley*, 2013].

At Titan, photoelectron escape may be expected to set up an ambipolar electric field as in the polar wind at Earth, driven in the simplest case by the average electron energy. This will be a similar situation compared to the open field lines in Earth's polar regions. Our observed upper limit is consistent with this when taking the full observed ionospheric electron spectra into account (see Figure 3).

**Figure 4:**
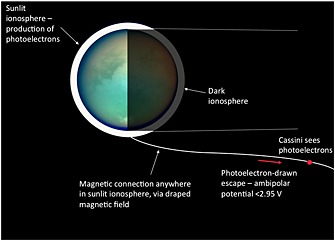
Schematic illustration of the magnetic connection between the sunlit ionosphere (left) and Cassini in the tail.

Our measurements at Titan suggest that in this case the field‐aligned potential has an upper limit of 2.95 V up to 6.8 *R*_T_—a similar result to the terrestrial case [*Coates et al*., 1985]. It is interesting to note that, as discussed earlier, the average electron kinetic energy should control any parallel ambipolar potential. This is consistent with the spectra shown in Figures 1, 2, 3. Although the 24.09 eV peak provides the unique identification of ionospheric photoelectrons in Titan's environment, the bulk energy of the observed electrons sets up the potential which we find is less than 2.95 eV Although some modelers have studied polar wind‐like effects at Titan [e.g., *Gan et al*., 1992, 1993; *Keller and Cravens*, 1994], the full photoelectron spectrum and field‐aligned potential is not always considered. We suggest that further modeling comparisons should now be made at Titan based on these results. Figure 4 summarizes the geometry of the observations.

The escape energy from Titan's ionosphere is <1 eV, so this process may be significant for atmospheric escape, although as the upper limit is 2.95 eV this will eventually limit the escape via this process. A review of the various thermal and nonthermal escape processes at Titan was given by *Johnson et al*. [2010], and estimates were provided by *Coates et al*. [2012].

## 6 Summary and Conclusions

In summary, we have presented new observations of the photoelectron spectra in Titan's sunlit ionosphere and in the tail. The results again confirm a magnetic connection between the sunlit ionosphere and tail, along the draped magnetic field lines [*Coates et al*., 2007a; *Wellbrock et al*., 2012].

Our new observation in this paper is that the ionospheric photoelectron peak occurs in the same ELS energy bin in both the sunlit ionosphere and in the distant tail. We assume that the spacecraft potential is similar in similar (ionospheric) ambient conditions, which is reasonable by comparison with other Cassini data sets [*Wahlund et al*., 2005]. We therefore find an upper limit for the ambipolar field‐aligned potential at Titan, between the sunlit ionosphere and up to 6.8 *R*_T_ in the tail, of 2.95 V.

We remark that this is at the lower end of estimates made for the polar wind at Earth and compares well with some terrestrial observations [*Coates et al*., 1985]. A polar wind style mechanism was reported at Titan [*Coates et al*. 2007a, 2011], which has led to escape rate estimates via this mechanism and others [*Coates et al*., 2012]. Clearly, the magnitude of the field‐aligned potential limits the overall escape flux from this process. To our knowledge, this is the first determination of an upper limit for a polar wind related ambipolar potential beyond Earth.
